# Antimutagenic Effect of* Hibiscus sabdariffa* L. Aqueous Extract on Rats Treated with Monosodium Glutamate

**DOI:** 10.1155/2017/9392532

**Published:** 2017-01-19

**Authors:** Ana Carla Guidini Valentini Gheller, Jacqueline Kerkhoff, Gerardo Magela Vieira Júnior, Kleber Eduardo de Campos, Marina Mariko Sugui

**Affiliations:** ^1^Institute of Natural, Humanities and Social Sciences, Federal University of Mato Grosso, Sinop, MT, Brazil; ^2^Department of Chemistry, Federal University of Piauí, Teresina, PI, Brazil; ^3^Institute of Biological and Health Sciences, Federal University of Mato Grosso, Barra do Garças, MT, Brazil; ^4^Institute of Health Sciences, Federal University of Mato Grosso, Sinop, MT, Brazil

## Abstract

*Hibiscus sabdariffa* L. is a plant of the Malvaceae family, commonly known as roselle.* H. sabdariffa* is known to contain antioxidant, cholesterol-lowering, antiobesity, insulin resistance reduction, antihypertensive, and skin cancer chemopreventive properties. This study evaluated the effects of* H. sabdariffa* aqueous extract against cyclophosphamide (CPA, 25 mg/Kg) induced damage to DNA in male Wistar rats by micronucleus test. Samples of* H. sabdariffa* calyx were obtained in the municipality of Barra do Garças, Mato Grosso, Brazil. The aqueous extract was prepared by infusion and each animal received a daily dose of 400 mg/Kg by gavage for 15 consecutive days of treatment. The presence of anthocyanins was confirmed by ferric chloride test and phenolic compounds using high-performance liquid chromatography, with emphasis on the identification of rutin. The animals were sacrificed by deepening of anaesthesia to obtain bone marrow and determination of the frequency of micronucleated polychromatic erythrocytes. The group treated with the aqueous extract of* H. sabdariffa* revealed a 91% reduction in micronucleus frequency when compared with the positive control group. Under the conditions tested,* H. sabdariffa* L. presented a protective effect to CPA-induced damage to DNA of the treated animals, and it is a potential candidate as a chemopreventive agent against carcinogenesis.

## 1. Introduction

*Hibiscus sabdariffa* L. is a plant of the Malvaceae family. It is a vigorous shrub which can reach up to three metres in height and can have a green or reddish stem. It has pink, purple, or creamy white flowers, with red or white fleshy calyces that form the fruits [1]. It is a tropical plant native to India and Malaysia, although it grows widely in the tropical and subtropical regions of both hemispheres [2]. The nutritional benefits of the plant are associated with the bioavailability of flavonoids, particularly anthocyanins [3].

Therapeutic properties of* H. sabdariffa* found in the literature include antioxidant [4], hypocholesterolemic [5, 6], antiobesity [7], insulin resistance reduction [6], antihypertensive [8], skin cancer chemopreventive [9], diuretic [10], antimicrobial, and cytotoxic properties [11]. Additionally, it can be pointed out that* H. sabdariffa* is used as a diaphoretic, mild laxative, and sedative in the treatment of kidney stones and liver injury [12].

The therapeutic properties of* H. sabdariffa* can be attributed to the presence of phenolic compounds-secondary metabolic products of this plant, known for their antioxidant potential [13]. Olaleye [14] found cardiac glycosides, flavonoids, saponins, and alkaloids in the aqueous extract of* H. sabdariffa*.

A wide variety of phenolic substances, especially those present in food and medicinal plants, have been considered as adjuvants in the prevention of genotoxic effects. Most of these naturally occurring phenolic compounds contain antioxidant and anti-inflammatory properties [13].

Substances known as chemopreventive are those capable of inhibiting, slowing, or reversing carcinogenesis at various stages [15]. Anthocyanins act as antioxidants and are therefore able to fight mutagenic processes [16].

Mutagenesis has become a sought-after subject of studies not only in Brazil, but worldwide. The relevance of this observation was due to the association between mutation and cancer. Ministry of Health data shows that, in 2013, 189.454 people died as a result of malignant neoplasms. For 2016, the occurrence of more than 596,000 cases of the disease in the country is estimated [17].

Given that mutation is an initiating event of cancer, it can be said that battling or preventing mutagenic events is one of the paths that should be developed further in an attempt to prevent damage to the genetic material [18]. According to Lippman and Hong [19], prevention research must increasingly integrate genomic studies with studies related to environmental factors, lifestyle, and diet.

According to the World Health Organization [20], obesity and overweight are defined as abnormal or excessive accumulation of fat, derived from a severe metabolic disorder. As of 2014, 52,5% of the adult population in Brazil were overweight [21]. This condition is a health risk linked to serious nutritional problems, which increase the risk of morbidity of various metabolic and chronic diseases among these neoplasms [22].

Traditional knowledge is an important source of information in locating and obtaining new herbal medicines. An ethnobotanical survey conducted by Bieski et al. [23] in a district of the city of Poconé, Mato Grosso, Brazil, presented* H. sabdariffa* as one of the most important plants used for medical purposes, which suggests that it is widely used in the region.

According to Ruiz et al. [24], there are still new plant species emerging with the potential to become sources of new drugs. However, popular and even traditional use is not enough to validate a plant as herbal medicine, since the lack of reliable information about its properties, adverse reactions, synergistic action, and toxicity is worrisome factor in self-medication [25].

In this context, this study aims to evaluate the effects of* H. sabdariffa* against DNA damage in obese male Wistar rats by means of the micronucleus test.

## 2. Materials and Methods

### 2.1. Animals

This study was approved by the Ethics Committee for Animal Research (CEUA/UFMT) by protocol number 23108.705702/13-9. Male Wistar rats which had received obesity inducing treatment with monosodium glutamate, according to the procedure of Campos et al. [26]. Confirmation of obesity was determined by Lee obesity index.

During the experimental period, the animals were kept in plastic cages in an experimental room of LiPeQ/UFMT/Sinop, under controlled conditions of temperature (22 ± 2°C), relative humidity (55 ± 10%), light cycle (12 hours light/dark), and exhaustion. They also received a pelleted commercial diet (Purina) and filtered water ad libitum.

### 2.2. Chemical Agent

Cyclophosphamide (CPA) is an alkylating agent that acts by blocking the replication of DNA. CPA (Baxter) was diluted in a 0.9% saline solution and administered intraperitoneally to the animals at a concentration of 25 mg/Kg body weight (b.w) in accord with Delmanto et al. [27].

### 2.3. Preparation of Extract

Samples of* Hibiscus sabdariffa* L. calyxes (740 g) were obtained in the municipality of Barra do Garças, Mato Grosso, Brazil. The plant voucher specimen was placed in the Botanical Garden of Brasilia, under record HEPH5633. An infusion was prepared with the already dried calyxes in a proportion of 15.0 g of vegetal material per 100 mL of water. The infusion was filtered and a sample of 5.0 mL was placed in an evaporation oven for dry weight determination. Each animal received a daily dose of 400 mg/Kg by gavage during treatment.

### 2.4. Identification of Anthocyanins

The test was based on the methodology of Mouco et al. [28], where any flavonoid compounds present within the sample will develop color in the presence of ferric chloride. Coloration may vary from green to yellow-brown and violet. To verify the presence of these compounds, two drops of ferric chloride 2.5% were added to an aliquot of 1.0 mL of concentrated crude extract.

### 2.5. High-Performance Liquid Chromatography (HPLC)

The solutions for HPLC analysis were prepared at a concentration of 10 mg·mL^−1^ in ultrapure water and filtered through a Millex® filter with a 0.45 *μ*m pore size. Chromatographic tests were performed on a Varian HPLC system 325 Pro Star LC plus UV detector with dual wavelength system. The chromatographic separation was performed on a reverse phase column Phenomenex Luna C18 (250.0 mm × 4.6 mm, 5 *μ*m). The mobile phase consisted of an aqueous solution of 0.1% by volume trifluoroacetic acid (eluent A) and acetonitrile (eluent B) with the following elution gradient: 0–10 min, 10% B; 10-11 min, 20% B; 11–15 min, 70% B; 15–25 min, 10% B. The flow rate was 0.8 mL/min; the injection volume was 20 *μ*L, and UV detection at 254 and 360 nm.

### 2.6. Micronucleus Test (MNT)

The in vivo micronucleus test is widely used in genetic toxicology research, even as a standard test for evaluation and registration of new pharmaceutical products. It enables assessment of the clastogenic effects that damage the chromosome and aneugenic effects that induce aneuploidy or abnormal chromosome segregation. The test relies on assessing the frequency of micronucleated polychromatic erythrocytes after exposure to the chemical agent that is to be investigated [29].

The acquisition and preparation of slides of bone marrow erythrocytes for evaluating micronucleus rate (MN) followed the methodology proposed by MacGregor et al. [30]. For each animal, two slides were prepared and decoded in a blind test. We analysed 2.000 cells per animal in a light microscope, with an amplification of 1.000 times (immersion).

The percentage reduction in the frequency of MN was calculated according to Waters et al. [31] by using the following equation:

(1) %  of reduction=frequency of MN in A−frequency of MN in Bfrequency of MN in A−frequency of MN in C×100,

where* A* represents the group treated with CPA (cyclophosphamide),* B* represents the group treated with* Hibiscus sabdariffa* and CPA, and* C* represents the group treated with 0.9% NaCl (control).

### 2.7. Experimental Design

**Figure 1:**
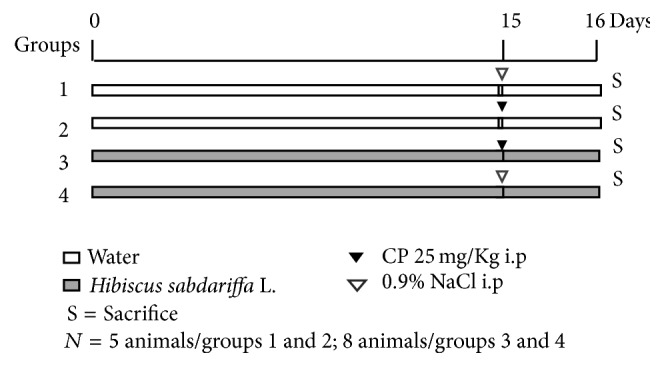
Experimental protocol for determination of antimutagenic/mutagenic effect of the* Hibiscus sabdariffa* L. in bone marrow cells of Wistar rats.

At 3 months of age, all animals were divided into four groups (*n* = 5 animals/groups 1 and 2; 8 animals/groups 3 and 4) in accord with the classification of obesity and treatment with* H. sabdariffa *aqueous extract (Figure 1): (1) control (treated with water and intraperitoneally injected with 0.9% NaCl on day 15), (2) cyclophosphamide (treated with water and intraperitoneal cyclophosphamide (25 mg/Kg b.w) on day 15), (3) treated with* H. sabdariffa* and intraperitoneal cyclophosphamide (25 mg/Kg b.w) on day 15, and (4) treated with* H. sabdariffa* and intraperitoneally injected with 0.9% NaCl on day 15.

Treatment was administered intragastrically (gavage) for 15 days at a dose of 400 mg/Kg/day b.w, adapted from Peng et al. [32]. Considering the weight gain of the animals during treatment, the dose was periodically adjusted. The amount of feed provided to the groups was weighed every day, making it possible to evaluate the average feed intake per animal.

After 15 days of treatment, animals were sacrificed by deepening of anaesthesia in order to obtain bone marrow for determining the frequency of micronucleated polychromatic erythrocytes (PCEs).

### 2.8. Statistical Analysis

The frequency of micronucleated cells in the different experimental groups was evaluated by chi-square test [33]. The significance test was *p* < 0.05.

## 3. Results

The test for the identification of anthocyanins was positive, generating a pronounced green coloration.

**Figure 2:**
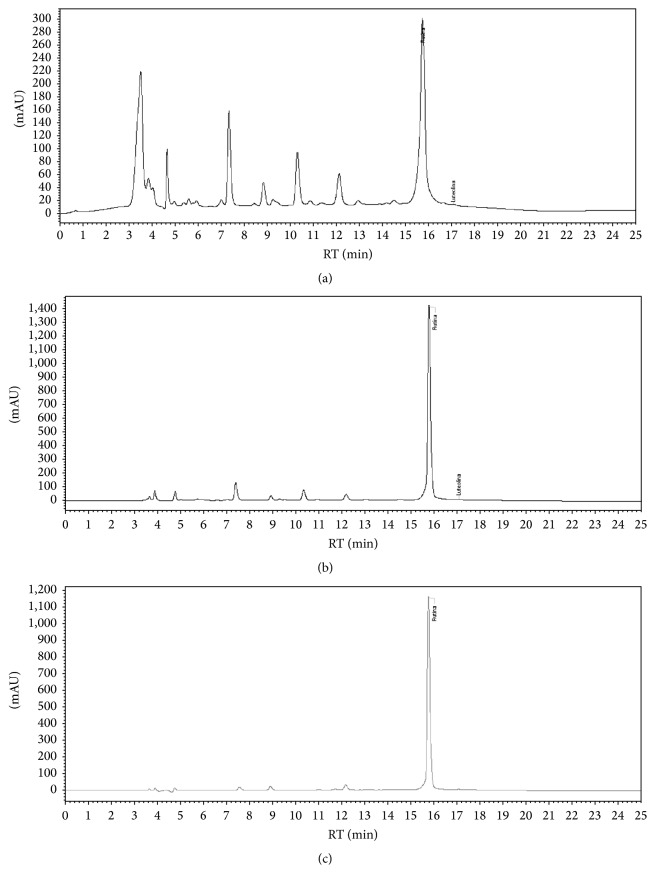
High-performance liquid chromatography (HPLC) of the aqueous extract of* Hibiscus sabdariffa*. (a) Coelution of* H. sabdariffa* extract, RT = 15.74 min 254 nm, (b) coinjection of standard rutin, RT = 15.78 min 254 nm and (c) rutin, RT = 15.74 min 360 nm.

The HPLC reading identified rutin at 254 nm (a) and 360 nm (c) levels, which were confirmed after the coinjection (b). It also indicates the presence of other phenolic compounds in the analysed extract (Figure 2).

Table 1 presents the effects of* H. sabdariffa* on cyclophosphamide-induced damage to DNA in rats pretreated with the aqueous extract. The results revealed that the group treated with* H. sabdariffa* aqueous extract and cyclophosphamide showed a reduction of 91% (*p* < 0.001) in the frequency of micronuclei in polychromatic erythrocytes in the bone marrow when compared to the positive control group. The results also showed that the group treated with only* H. sabdariffa* did not present any mutagenic potential when compared with the negative control group.

Table 2 shows the data of both the initial and final body weight of the rats, as well as the weight gain of the animals during the treatment period. It can be seen that the rats treated with* H. sabdariffa* L. aqueous extract had a lower weight gain when compared to the control groups.

Table 3 shows the average feed intake of each animal during the experimental period. According to the data, the groups treated with* H. sabdariffa* L. showed no significant difference in relation to feed intake when compared to the negative control group.

## 4. Discussion

The search for substances with anticancer properties derived from dietary plants currently plays an important role in preventing diseases. Many compounds present in natural products are considered chemopreventive agents-substances capable of counteracting toxicity to genetic material and consequent carcinogenesis. According to Lin et al. [15], the aqueous extract of* H. sabdariffa* exhibits chemopreventive properties that can contribute to the antimutagenic effects of the plant.

The results suggest that the use of* H. sabdariffa* as a medicinal plant brings benefits related to chemoprevention of DNA damage and subsequent prevention of cancer. The extract of* H. sabdariffa* reduced CPA-induced damage to bone marrow cells of male Wistar rats by 91% when compared with cyclophosphamide group.

Adetutu et al. [34] performed the micronucleus test in mice using sodium arsenite as an inducer of mutagenicity. The animals were treated with* H. sabdariffa* aqueous extract for 7 days at different concentrations: 50, 100, and 150 mg/Kg/day. The extract showed the potential for protection against damage induced by the chemical agent. The best results were found in the extract containing a concentration of 100 mg/Kg/day.

Rosa et al. [35] observed antigenotoxic and antimutagenic potential in the methanolic extract of* Hibiscus tiliaceus*, a plant that belongs to the same genus of* H. sabdariffa*, against oxidative mutagenesis induced by hydrogen peroxide and tert-butyl-hydroperoxide in cultured lung cell fibroblasts in a Chinese hamster (V79 cells) using the comet assay and micronucleus test. The results showed a reduction in the rate of DNA damage in the comet assay and it also showed a decrease in the mutagenic effect of the agents tested, verified by a reduction in the micronucleus rate.

Abdul Hamid et al. [36] identified antigenotoxic activity of* H. sabdariffa* aqueous extract in bone marrow cells of ICR mice with DNA damage induced by hydrogen peroxide using the comet assay. Cells were pretreated for 24 hours with 500 and 1000 ng/mL extracts and showed significant protection against hydrogen peroxide-induced damage.

Olvera-García et al. [37] observed antimutagenic activity of* H. sabdariffa *aqueous extract against the mutagenicity of 1-nitropyrene (1-NP) by way of a modified Ames test. It showed no genotoxic activity, since it did not induce DNA fragmentation in electrophoresis gel.

Some studies report the chemopreventive properties of* H. sabdariffa* by other action mechanisms. Lin et al. [38] evaluated the induction of apoptosis of* H. sabdariffa* aqueous extract against gastric cancer cells, concluding that it can be used as a chemopreventive agent. The feasibility of prostate cancer cell exposure to* H. sabdariffa* aqueous extract was also tested, and the inhibition of the growth of cells might be mediated via both intrinsic and extrinsic apoptotic pathways. The study showed an inhibitory effect on cell growth in a dose-dependent manner with a 50% inhibition of viability of cancer cells.

Adaramoye et al. [39] observed that the results of* H. sabdariffa *methanol extracts showed protective properties after exposure to radiation used to treat cancer. The study exposed male Wistar rats treated with* H. sabdariffa* to radiation and found that the consumption of the plant can be an excellent factor of protection against radiation-induced damage to the antioxidant system.

Thus, the chemopreventive effects observed in studies with* H. sabdariffa* are probably related to the antioxidants it contains. Chromatographic analysis (HPLC, Figure 2) of the aqueous extract confirmed that phenolic compounds—familiar antioxidants—were present in the infusion consumed by the animals. Among them were anthocyanins, confirmed by identification test with ferric chloride, and rutin, identified by HPLC and confirmed after standard coinjection.

Rodríguez-Medina et al. [16] performed a chemical characterization of* H. sabdariffa *aqueous extract using high-performance liquid chromatography (HPLC), which displayed 17 phenolic compounds. Anthocyanins are phenolic compounds of higher concentration in* H. sabdariffa* aqueous extract [40, 41].

According to the tests, anthocyanins were present within* H. sabdariffa* aqueous extract. The presence of these compounds in the extract is confirmed visually due to their intense purple color [10, 16].

The chromatographic analysis (HPLC) performed by Beltrán-Debón et al. [42], in which the first peaks to emerge in the chromatogram were identified as hydroxyl citric acid, hibiscus acid, and chlorogenic acid, showed a similar profile to the chromatogram presented in our results. There is a possibility that the obtained chromatogram also presents the same substances, as seen in the chromatograms (a), (b), and (c), where there are peaks not identified with low retention time and considerable concentration.

The quantification of anthocyanins performed by Johnson et al. [43] resulted in concentrations of 0.583 g ± 0.13 per 100 g of aqueous extract from* H. sabdariffa* calyxes. In the study by Alarcón-Alonso et al. [10], the concentration of anthocyanins in the extract was 77.3 mg/g, quercetin 3.2 mg/g, rutin 2.1 mg/g, and chlorogenic acid 2.7 mg/g.

Sindi et al. [44] isolated four types of anthocyanins in the aqueous extract of* H. sabdariffa*. The relationship between antioxidant activity and concentration of anthocyanins obtained from* H. sabdariffa* calyxes was determined by Tsai et al. [3], who concluded that the antioxidant capacity of the extract increases linearly with an increasing number of petals used in preparing the extract, as well as extraction time.

However, due to the lack of analytical standards, it was only possible to confirm the identity of rutin in the study, but it is possible that there may be other phenolic compounds in the chromatogram, considering favourable analysis conditions for the isolation thereof. Lin et al. [38] also isolated rutin in the aqueous extract of* H. sabdariffa*.

The study by Olvera-García et al. [37] showed that* H. sabdariffa* aqueous extract subjected to HPLC showed a higher concentration of substances when compared to chloroform and ethyl extracts investigated under the same conditions, suggesting that, for many compounds, extraction in an aqueous solution would be the preferable method.

The antioxidant activity of* H. sabdariffa* extract was evaluated by Tseng et al. [9], by the extinction capacity of 1-diphenyl-2-picrylhydrazyl (DPPH) and activity inhibition of xanthine oxidase (XO). The extract showed the ability to fight free radicals and showed a strong inhibitory effect on XO activity. The authors also tested the antioxidant bioactivity of these extracts using a tert-butyl hydroperoxide model (t-BHP), capable of inducing oxidative damage in the primary hepatocytes of rats. The results showed the potential of* H. sabdariffa* extract in protecting rat hepatocyte cytotoxicity induced by t-BHP and genotoxicity by different mechanisms.

Hepatoprotective effects were observed against chronic liver fibrosis induced by chemicals in vivo, closely associated with the antioxidant power of* H. sabdariffa *[45]. Also, Liu et al. [46], demonstrated a hepatoprotective effect in rats after exposing the animals to acetaminophen (paracetamol). Animals treated with* H. sabdariffa* aqueous extract showed a reduction in oxidative stress and liver cell death.

A study by Ademiluyi et al. [47] investigated the protective effect of* H. sabdariffa *on nephrotoxicity and oxidative stress in rats. The comparison between groups showed that the consumption of diets supplemented with the extract protects kidneys and reduces oxidative stress by modulation of antioxidants, especially anthocyanins.

Regarding the toxicity of* H. sabdariffa*, according to the results obtained, its aqueous extract showed no mutagenic activity when animals treated only with* H. sabdariffa* were compared to the control group. Chewonarin et al. [48] also observed that* H. sabdariffa* is not a mutagenicity inducer by means of a study performed via mutagenicity test in* Salmonella typhimurium* (Ames test).

Moreover, Takeda and Yasui [49] reported the mutagenicity of* H. sabdariffa*, suggesting the quercetin compound was responsible for mutagenic action. Montero et al. [50] identified mutagenicity of* Hibiscus elatus *Sw in rodent bone marrow by means of micronucleus test. Magenis et al. [51] observed genotoxicity of* Hibiscus acetosella* extract in peripheral blood and liver tissue of Swiss mice by comet assay.

Olaleye [14] found that* H. sabdariffa* was toxic to* Artemia salina* with CL50 of 55.1 ppm. Additionally, Al-Mamun et al. [11] observed cytotoxic effects of* H. sabdariffa* aqueous extract in* Artemia salina*, using vinblastine sulphate as a positive control. Orisakwe et al. [52] identified that the aqueous extract of* H. sabdariffa* calyx induces testicular toxicity in rats.

Preliminary studies (not shown) suggest antitumor activity of* H. sabdariffa* aqueous extract in a study where the cytotoxic effects were observed for Ehrlich Ascites Tumor cells (EAT) treated with concentrations of 1 mg/mL, 0.5 mg/mL, and 0.25 mg/mL extract. Furthermore, Olvera-García et al. [37] observed cytotoxic activity on human cervical carcinoma cells (HeLa) in vitro.

Many studies have reported the pharmacological effects of* H. sabdariffa* as antiobesity agent [7, 53]. Results revealed that groups treated with* H. sabdariffa* aqueous extract showed less weight gain when compared to control and cyclophosphamide groups. Alarcon-Aguilar et al. [7] used MSG as an obesity inducer and observed a significant reduction in weight gain of animals treated with 120 mg/Kg/day of* H. sabdariffa* aqueous extract. Iyare et al. [54] performed weight control in obese rats not treated with* H. sabdariffa* aqueous extract and witnessed a lower weight gain when compared to the negative control.

Pérez-Torres et al. [55] recommend the therapeutic use of* H. sabdariffa *extract in patients with metabolic syndrome due to polyphenol action. Peng et al. [32] identified the protective effect of polyphenol in* H. sabdariffa* extract against type II diabetes in an animal model at a dose of 200 mg/Kg. Therefore, future studies listing* H. sabdariffa* and metabolic disorders must be performed, as well as in relation to its use against other diseases.

## 5. Conclusion

In conclusion, under the experimental conditions used in this study,* H. sabdariffa* L. showed potential chemoprotective properties against DNA damage, probably contributing to the fight against free radicals. According to Ferreira et al. [56], DNA chemoprotective activity may be associated with phenolic compounds acting as potential antimutagenic agents, due to antioxidant action.

Experimental evidence correlating the ingestion of* H. sabdariffa* L. and mutation frequency are still very limited. However, the study showed that* H. sabdariffa* L. contains antimutagenic compounds, indicating it has potential chemopreventive properties against genotoxic substances, consequently preventing carcinogenesis.

The study also showed satisfactory results in the use of* H. sabdariffa* aqueous extract in the control of body weight. However, its consumption should be performed with caution, since there are still many therapeutic effects of* H. sabdariffa* to be investigated.

## Figures and Tables

**Table 1:** The effect of *H. sabdariffa* L. on the frequencies of MNPCEs in the bone marrow after treatment with cyclophosphamide (CP).

Treatment	Number of analyzed cells	MNPCEs		Reduction (%)
		No	%	
Water + 0.9% NaCl^a^	10.000	83	0.83	
Water + CP (25 mg/Kg)^b^	10.000	128	1.28	
*H. sabdariffa* L. + CP (25 mg/Kg)	16.000	87	0.87	91^*∗*^
*H. sabdariffa* L. + 0.9% NaCl	16.000	60	0.60	

^a^Control; ^b^Cyclophosphamide; ^*∗*^*p* < 0,001. Chi-square test.

**Table 2:** Mean body weight (g) and weight gain (mean ± SD) for rats treated for 15 days with aqueous extract of *H. sabdariffa* L.

Treatment	Number of animals	Initial weight (g) *X* ± SD	Final weight (g) *X* ± SD	Weight gain (g) *X* ± SD
Water + 0.9% NaCl^a^	5	220.37 ± 32.66	273.0 ± 28.63	48.4 ± 23.98
Water + CP (25 mg/Kg)^b^	5	201.87 ± 37.79	273.0 ± 4.47	60.0 ± 32.59
*H. sabdariffa* L. + CP (25 mg/Kg)	8	203.11 ± 26.26	236.25 ± 28.75	35.52 ± 10.83
*H. sabdariffa* L. + 0.9% NaCl	8	207.87 ± 31.74	248.12 ± 38.53	40.25 ± 27.44

^a^Control; ^b^Cyclophosphamide. Mean and standard deviation.

**Table 3:** Average feed intake and *H. sabdariffa* L. (dry weight) for rats treated for 15 days with the aqueous extract of *H. sabdariffa* or water.

Treatment	Number of animals	Body weight (g) *X* ± SD	Feed consumption (g/day/animal) *X* ± SD	Consumption of *H. sabdariffa* (mg/animal/day)
Water + 0,9% NaCl^a^	5	248.8 ± 29.82	22.01 ± 3.43	—
Water + CP (25 mg/Kg)^b^	5	243.0 ± 34.69	24.30 ± 2.55	—
*H. sabdariffa* L. + CP (25 mg/Kg)	8	218.4 ± 25.55	22.88 ± 1.42	87.37
*H. sabdariffa* L. + 0,9% NaCl	8	228.0 ± 30.42	20.87 ± 2.21	91.20

^a^Control; ^b^Cyclophosphamide. Mean and standard deviation.
